# Mapping stress inside living cells by atomic force microscopy in response to environmental stimuli

**DOI:** 10.1080/14686996.2023.2265434

**Published:** 2023-10-18

**Authors:** Hongxin Wang, Han Zhang, Ryo Tamura, Bo Da, Shimaa A. Abdellatef, Ikumu Watanabe, Nobuyuki Ishida, Daisuke Fujita, Nobutaka Hanagata, Tomoki Nakagawa, Jun Nakanishi

**Affiliations:** a Research Center for Macromolecules and Biomaterials, National Institute for Materials Science, Tsukuba, Ibaraki, Japan; b Center for Basic Research on Materials, National Institute for Materials Science, Tsukuba, Ibaraki, Japan; c Research Network and Facility Services Division, National Institute for Materials Science, Tsukuba, Ibaraki, Japan; d Department of Diagnostic Pathology, University of Tsukuba Hospital, Tsukuba, Ibaraki, Japan

**Keywords:** Cellular prestress, AFM indentation, nucleoskeleton mechanics, informatics AFM, tensegrity

## Abstract

The response of cells to environmental stimuli, under either physiological or pathological conditions, plays a key role in determining cell fate toward either adaptive survival or controlled death. The efficiency of such a feedback mechanism is closely related to the most challenging human diseases, including cancer. Since cellular responses are implemented through physical forces exerted on intracellular components, more detailed knowledge of force distribution through modern imaging techniques is needed to ensure a mechanistic understanding of these forces. In this work, we mapped these intracellular forces at a whole-cell scale and with submicron resolution to correlate intracellular force distribution to the cytoskeletal structures. Furthermore, we visualized dynamic mechanical responses of the cells adapting to environmental modulations in situ. Such task was achieved by using an informatics-assisted atomic force microscope (AFM) indentation technique where a key step was Markov-chain Monte Carlo optimization to search for both the models used to fit indentation force–displacement curves and probe geometry descriptors. We demonstrated force dynamics within cytoskeleton, as well as nucleoskeleton in living cells which were subjected to mechanical state modulation: myosin motor inhibition, micro-compression stimulation and geometrical confinement manipulation. Our results highlight the alteration in the intracellular prestress to attenuate environmental stimuli; to involve in cellular survival against mechanical signal-initiated death during cancer growth and metastasis; and to initiate cell migration.

## IMPACT STATEMENT

Quantitative knowledge of internal force distribution in living cells is crucial to understand how cellular machinery responds mechanically to changes in the extracellular environment. Conventional force-sensing methods, which rely on pre-attached sensors, are incapable of providing whole-cell information with high spatial resolution. By machine learning assistance, we could improve AFM indentation’s force sensitivity to a level that now enabled force (prestress) to be mapped on whole living cells with submicron resolution. The new capability revealed that cells actively redistribute forces among intracellular components to mitigate negative influence from the environment, and such response mechanisms could be used to understand ailing cellular states like cancer.

## GRAPHICAL ABSTRACT

**Figure:**
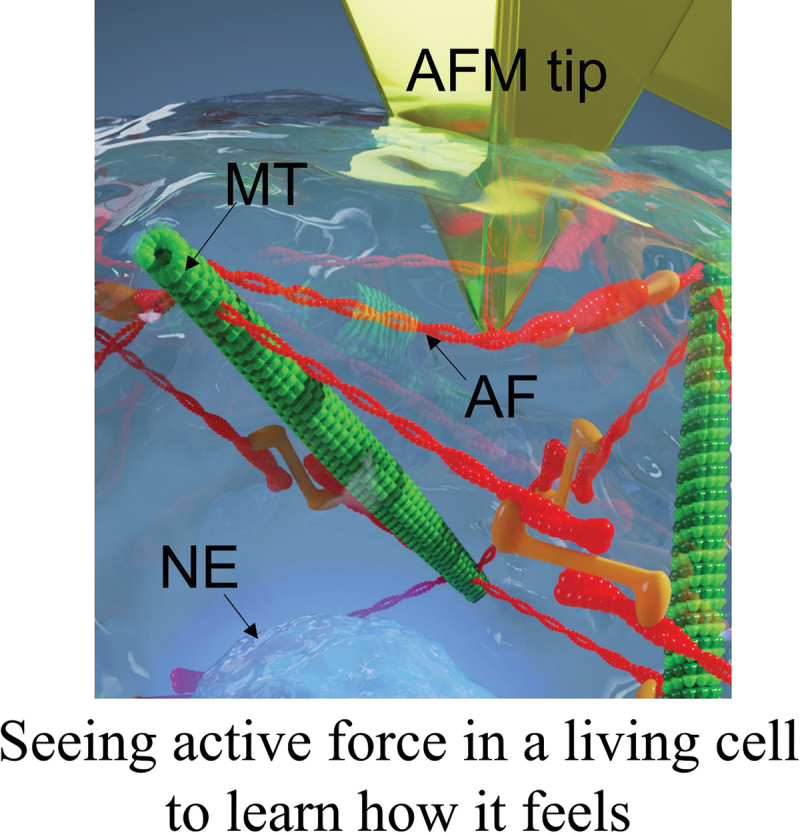

## 1. Introduction

Cells are exposed to both chemical and physical stimuli from extracellular environment, as random disturbances or intentional signal inputs to direct cellular phenotypes. Such responses fall into two categories: either change for the cell to adapt to the stimuli; or programmed cell death when the stimuli exceed the adaptability threshold [1]. Under physiological and pathological conditions, effective execution of such cellular feedback mechanism is crucial to maintain homeostasis at the organ level [2]. On the other hand, it is evident that a flawed cellular response is associated with many challenging human diseases, including diabetes, Parkinson’s disease, myocardial infarction and cancer [1]. Cellular responsive actions were physically implemented by active forces exerted on each cellular component. These forces, while individually acting on different intracellular locations with various magnitudes and directions, also coordinately form a cellular tensegrity that keeps cell shape and stiffness [3]. Therefore, measuring the complex distribution of intracellular forces within a high resolution is essential for a mechanistic understanding of cellular responses to environmental changes.

Although micro-rheology methods [4] and Förster resonance energy transfer-based probes [5] were used to measure intracellular forces where sensors were pre-attached, they could not ubiquitously cover a cell where the whole cellular tensegrity extends. Other methods capable of 3D tissue study lack of high spatial resolution to access individual molecular machinery [6]. In sharp probe AFM indentation, a nanometric tip was scanned over an area covering a whole cell [7]. The resistance force acting on the AFM tip contains contribution from prestress [8]. Because in each indenting position, the tip is pressed into cell surface and interacts with cellular components underneath, the collected force–deformation (*f-d*) curves also allow depth-dependent analysis. However, to extract correct stress (force per area) information from the collected *f-d* curves, one must know the correct analytical model to fit with indentation data and knowledge of the correct indenter tip shape [9]. For conventional specimen with flat surface and uniform composition, high-fidelity numerical simulation could be used to verify if a selected model and associated tip descriptors could reproduce experimental data [10]. However, in the case of a living cell, the surface topography is complex; internal structure and composition were both unknown. Therefore, it is not feasible to build a simulation model to represent real structure and real mechanical property of a living cell. For cell specimen, directly applying conventional indentation models built with flat surface assumption could also lead to topography induced artifact [11]. In this work, we factorized possible influences from model selection, topography and indentation depth into a multivariate mode. We then optimized these variates using Markov-chain Monte Carlo (MCMC) method toward the lowest accumulative fitting error for all *f-d* curves collected from the same single cell [12]. The resultant model enabled quantifying both prestress and elastic modulus of a whole cell tensegrity with submicron resolution. With time-lapse snapshots, we revealed how intracellular force distribution was manipulated when the cell is influenced by a few selected chemical and physical stimuli, which were intentionally induced in extracellular environment.

## 2. Results and discussion

### 2.1. Mapping prestress distribution in cellular tensegrity using informatics-assisted AFM

**Figure 1.**
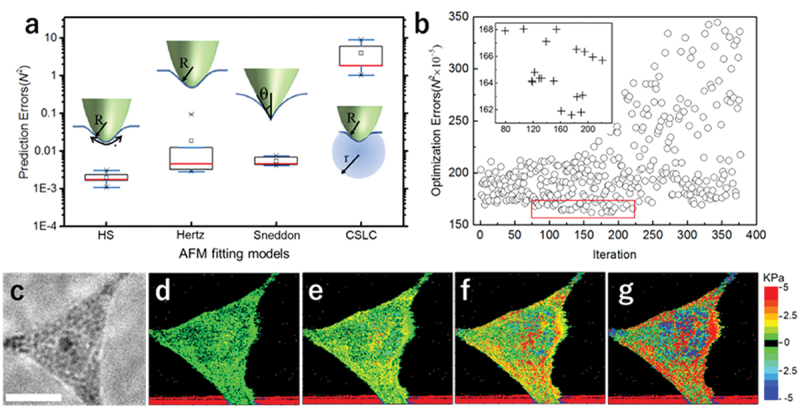
Prestress distribution measurement by informatics-assisted AFM. (a) Prediction error comparison using four popular AFM fitting models: horizontal prestress (HS), Hertz model (H), Sneddon model (sne) and cortical shell with liquid core model (CSLC). (b) Optimization error produced during Markov-chain Monte Carlo search for the most suitable combination of AFM probe shape descriptors. (c) Phase contrast image of a Hela cell. Scale bar: 15 μm. (d)–(g) Four representative AFM prestress maps of the Hela cell produced as probe shape descriptors being gradually optimized.

We began with four popular fitting models that have been applied for AFM indentation on cell specimen. Those include the three Hertzian models with spherical tip (Hertz model), conical tip (Sneddon model) and spherical tip with additional force contribution from horizontal prestress (HS model) [13–15]. The fourth is a non-Hertzian model which considers cell structure as a cortical shell with liquid core (CSLC model) [16]. For all models, the influences of substrate, cell topology and indentation depth were considered equally with the same treatments, as listed in Table 1 [17]. Tip radius, R, was expressed as a linear combination of a constant term r, two topographical gradient-dependent terms of *a δz/δx* and *b δz/δy* and a depth-dependent term of *c d*, where *a, b, c* are to-be-determined coefficients (Supplementary information, Figure S1). Each model thereby includes four tip radius variates, *r, a, b, c*, and model-specific variates, such as Young’s modulus, *E*, in Hertzian models. A spectrum image, which is defined as a 2D matrix of 256 × 256 *f-d* curves was acquired on a living Hela cell. For a given set of tip radius variates, model variates were determined by linear regression fitting with experimental data. The produced minimum fitting error was used as feedback to a MCMC algorithm to optimize the tip radius variates (Supplementary information, Figure S2). Experimental data were further divided into training group (7/8) and test group (1/8), where all variates determined by training group were used to predict *f* values from *d* values measured in the test group. The difference between such predicted *f* value and experimentally measured *f* value thus formed a prediction error (Supplementary information, Figure S3). Figure 1(a) shows a comparison of prediction errors obtained from all four models. The result indicated that HS model best simulates the true cell indentation scenario with the lowest prediction error. One cycle of MCMC optimization toward the lowest fitting error for the HS model is shown in the plot of Figure 1(b). Prestress maps generated in the iteration producing the lowest fitting error and the preceding three iterations are displayed in Figure 1(d–g), with Figure 1(c) being phase contrast image of the cell. It shows that as fitting error becomes lower, contrast of fine features in the stress maps becomes higher. Prestress-related term in HS model is treated as an addition to the classical Hertz model, which is similar to the definition of fitting error using Hertz model. It suggested that such prestress information would be overlooked as fitting errors if classic Hertz model was used. It was noted that map of gradient in the scanning direction (*δz/δy*) contributes the most significantly to the final optimized R distribution (Supplementary information, Figure S5). It is likely due to the synchronization error between AFM scanner lateral movement (scanning) and tip vertical movement (indentation). Such error, though not previously aware of, was accounted for by the MCMC optimization for *R* value.

**Table 1.** Force and tip shape expressions used in the four evaluated models.

Model	Force expression	Tip shape expression
HS	$\begin{array}{rl} & F=4\pi Rd\sigma cos\left[tan^{-1}\left(2\sqrt{\frac{R}{d}}\right)\right]\\ & +\frac{4}{3\left(1-\gamma^{2}\right)}E\sqrt{R}d^{1.5} \end{array}$*R*: tip radius; *σ*: prestress; *d*: deformation; g: Poisson’s ratio; *E*: Young’s modulus	$R=r+a\frac{\partial z}{\partial x}+b\frac{\partial z}{\partial y}+cd$
Hertz	$F=\frac{4}{3\left(1-\gamma^{2}\right)}E\sqrt{R}d^{1.5}$	$R=r+a\frac{\partial z}{\partial x}+b\frac{\partial z}{\partial y}+cd$
Sneddon	$F=\frac{2tan\theta }{\pi \left(1-\gamma^{2}\right)}Ed^{2}$*q*: tip half angle	$\theta =\theta_{0}+a\frac{\partial z}{\partial x}+b\frac{\partial z}{\partial y}+cd$
CSLC	$F=4T\left(\frac{1}{r}+\frac{1}{R}\right)\pi Rd$*T*: cortical tension; *r*: cell radius	$R=r+a\frac{\partial z}{\partial x}+b\frac{\partial z}{\partial y}+cd$

**Figure 2.**
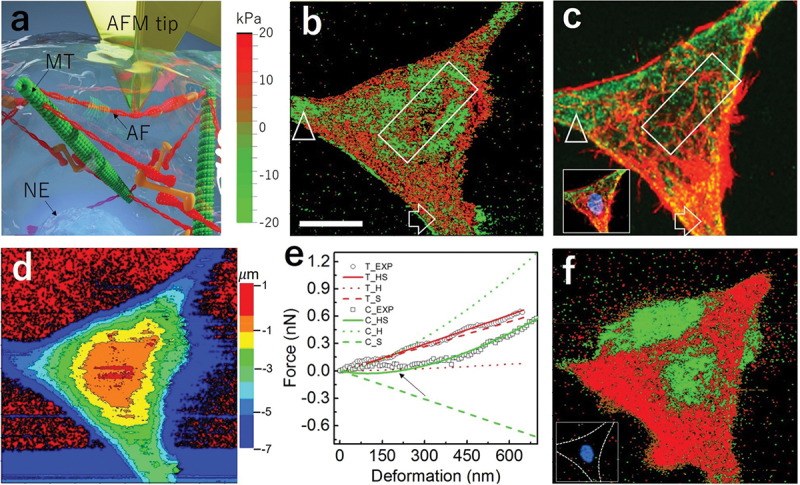
Correlation between AFM prestress map and cytoskeletal structure. (a) Illustration explaining how AFM probe senses prestress existing on cellular tensegrity, which is composed of actin fiber (AF), microtubule (MT) and nucleus envelope (NE). (b) A living Hela cell prestress map showing distribution of tensile stress (red) and compressional stress (green). Scale bar: 10 μm. (c) fluorescence image of cytoskeleton showing actin fiber (red) and microtubule (green). Inset: same image including nucleus (blue). (d) AFM topography map. (e) Two typical AFM force–deformation curves with fitting using HS model, when prestress is tensile (red) or compressional (green). Stress fitting components (S) and modulus fitting components (H) were also plotted. (f) Prestress map of the same Hela cell after chemical fixation. Inset showing nucleus position.

The physical meaning of HS model applied to Hela cell indentation was elucidated using an illustration of Figure 2(a) based on the tensegrity model [3]; actin fibers (red) are stretched toward each other by the activities of myosin motors (orange). The tensional-stressed actin fibers thus result in compressional stress in the microtubules (green) which they are tied to. Therefore, when an AFM probe is pressed onto an actin fiber, two types of forces are expected to act on the probe tip: countering force due to elastic deformation of the fiber and upward partial component of the pre-existing tensional stress. Prestress map using HS model is presented again in Figure 2(b) using a two-color theme for tensional stress (red) and compressional stress (green). After spectrum image acquisition, the living Hela cell specimen was immediately fixed with formaldehyde. Another spectrum image was then acquired on the same cell after chemical fixation within 20 minutes. It guaranteed the same AFM instrument conditions with the previous image. The fixed cell specimen was then stained with Hoechst, anti-tubulin and phalloidin to visualize nucleus, microtubules and actin fibers, respectively. The fluorescence image displayed in Figure 2(c) represents microtubule (green) and actin fiber (red), while the inset showed a merged image together with nuclear staining (blue). It is interesting to note that Figure 2(b) and c shows a good correlation between cytoskeletal structure distribution and prestress distribution, with tensional stress born by actin fiber (arrow) and compressional stress born by microtubule (arrowhead). A region showing a thick stress fiber is marked by a rectangle in Figure 2(c). In the equivalent region of Figure 2(b), high tensional stress was also found extending in the same direction and length as the stress fiber. These results agree well with cell tensegrity theory which states that myosin II motors create tension in actin fibers through ATP hydrolysis and such tension was balanced by compressional stress passively carried by microtubules [3]. Figure 2(d) is a simultaneously collected topography map, where a height gradient is found centering around the nucleus. It is important to note there is no strong correlation between the prestress map and the topography map even though they were extracted from the same force spectrum data. It verified that the observed prestress distribution is not an artifact induced by topography variation, but closely related to internal cytoskeleton distribution. Two typical indentation curves collected from the tensional region (arrow) and compressional region (arrowhead) in Figure 2(b) are plotted in Figure 2(e). Fitting curves produced by HS model were also plotted together with experimental data for comparison. In Table 1, we describe the force expression in HS model as summation of stress-related contribution and modulus-related contribution. Besides the force sum (HS), we also plotted these two force components (S, H) separately in the modified Figure 2(e). It is worth noting that in the shallow indentation range, negative total force is expected, as shown by the green solid line. It meant that compressional stress bearing parts in the cell would autonomously bend away from the tip and the AFM tip would feel no resistance when indenting downward. That might explain the force value returned to zero after initial contact, as marked by an arrow in the figure. It shows that the higher force predicted by HS model in the actin fiber region correctly accounted for the additional resistance force from tensional stress, while the lower force in the microtubule region suggested a lower resistance due to compressional stress contribution [18]. HS model can also be viewed as characterizing linear deviation from the classical Hertz model. When the linear term slope is positive, tensional stress is assigned and negative slope value corresponds to compressional stress. It must be emphasized that only when other influences are sufficiently removed can one attribute all deviation to stresses. Such influencing factor removal process was done by MCMC algorithm in this case. However, such cytoskeleton–stress correlation completely disappeared in the stress map for the same cell after chemical fixation (Figure 2(f)). Protein cross-linking stopped ATP hydrolysis of myosin II motors and consequently removed actin fiber tension. The force balance created in the living cell tensegrity network was destroyed upon fixation. The fact that the nucleus fluorescence image of Figure 2(f) inset, post-fixation stress map of Figure 2(f) and topography map of Figure 2(d) all share the same threefold symmetry supported the above speculation. This verified that the stress distribution observed in the living status is generated by biological activities of cells.

### 2.2. Nucleoskeletal tension increased to compensate cytoskeletal tension loss during myosin II inhibition

**Figure 3.**
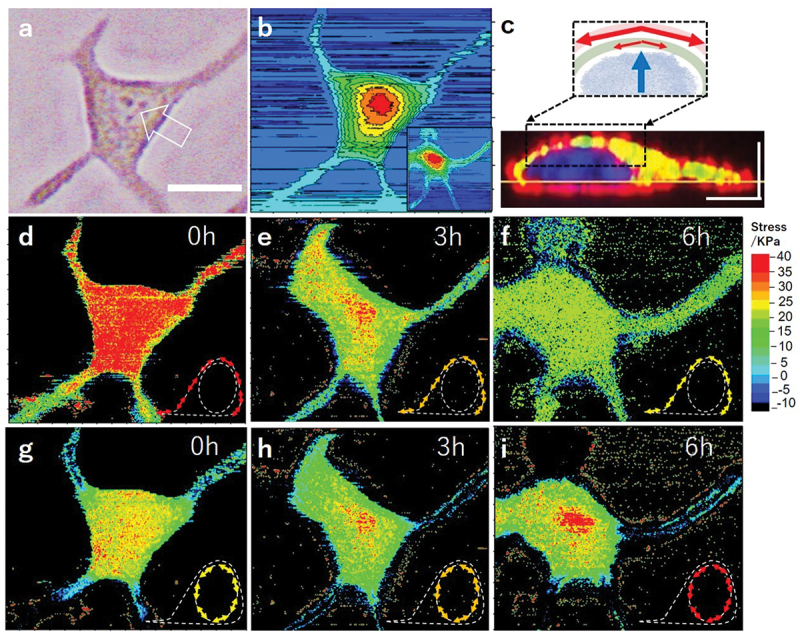
Prestress mapping on a living MCF7 cell during myosin II motor inhibition. (a) Phase contrast image of a MCF7 cell. Scale bar: 15 μm. (b) AFM topography map. Inset: topography map of the same cell 6 hours after myosin II inhibition. (c) Confocal fluorescence microscopy side view of a MCF7 cell, showing actin cortex (red), microtubule (green) and nucleus (blue). Scale bar: 5 μm in both vertical and horizontal dimension. Arrows showing internal forces, (d)-(f) A series of prestress maps of the MCF7 cell produced using indentation curves up to a depth of 100 nm, following a time sequence after myosin II inhibition. Insets showing data collected on cell cortex. (g)–(i) Same type of prestress maps using full depth of 800 nm. Insets showing data collected on nucleoskeleton.

Next, we studied force dynamics under Myosin II motor inhibition. Figure 3(a) is a phase contrast optical image of a human breast cancer cell (MCF7), for which nucleus position is marked by an arrow. AFM force spectrum image was acquired for this cell. The generated topography map is shown in Figure 3(b). As expected, the highest region on the cell coincides with the nucleus position. Confocal fluorescence images with stained nucleus, actin fiber and microtubule suggested that nucleus occupies majority of cell volume in this type of cells. A typical side view of fluorescence image is displayed in Figure 3(c), where blue, red and green were used to label nucleus, actin fiber and microtubule, respectively. Depth-dependent modulus and prestress maps were generated by using selected range of deformation from the *f-d* curves. Figure 3(d,g) are prestress maps with depths of 100 nm and 800 nm, which correspond to the prestress distributions at the outmost cortex and nucleoskeleton layers, respectively, as illustrated in the figure inset of each image. It is well known that modulus of cytoskeleton is decreased after cells treated with cytoskeletal destabilizing drugs—Blebbistatin [19]. Figure S4(a–c), respectively, shows cortical modulus maps before, 3 hours and 6 hours after an addition of 10 μM Blebbistatin into the culture media to block the myosin II motor activity. It is clearly seen that cortical modulus shows a downward trend with increase of time. Figures S4(d–f) are the corresponding modulus maps of nucleoskeleton before, 3 hours, and 6 hours after the blebbistatin treatment, respectively. It revealed that there was almost no significant change in nucleoskeletal modulus. This finding is consistent with the previous AFM study on the Young’s moduli of nucleus after different treatments [19]. However, as a result of myosin inhibition, cortical tension reduced (Figure 3(e) while the nucleoskeletal tension increased (Figure 3(h)). These results show for the first-time simultaneous observation of nuclear modulus and tension, and its clear difference between them, in response to the myosin inhibition. It is suggested that cell compensated the reduced cortical tension by increasing nucleoskeletal tension to maintain the mechanical balance within the cells. This happened regardless of the nucleus innate elastic properties. This suggested the contribution of nucleus to manipulate the cellular tension despite the absence of actomyosin. In addition, most of previous mechanical studies for nucleus requires the separation of nucleus [20] which overlook the intracellular mechanical balance necessary to maintain the cells integrity. Thus, our technique not only enabled the direct in situ observation of nuclear adaptive mechanical responses to the decrease in the cytoskeletal tension in living cells but also the detection was within a higher spatial resolution. By elongation of myosin II inhibition up to 6 hours, cortical tension dropped by 50% (from ~40 kPa to ~ 20 kPa (Figure 3(f)). At the same time, topography map (Figure 3(b) inset) showed cell volume increase of 50% compared to that before Blebbistatin addition. Figure 3(i) shows that nucleoskeletal tension has increased from ~ 30 kPa to ~ 40 kPa by 33%. This alteration in nuclear tension is expected to alter chromatin’s folding and deformation that is crucial in regulating cellular transcription [21,22]. This experiment therefore suggested a new mechanism, in the event of weakened cytoskeletal tension, the cellular hemostasis is maintained through controlling nucleoskeletal tension.

### 2.3. Cancer cells manipulate stress distribution differently from normal cells under compressional force

**Figure 4.**
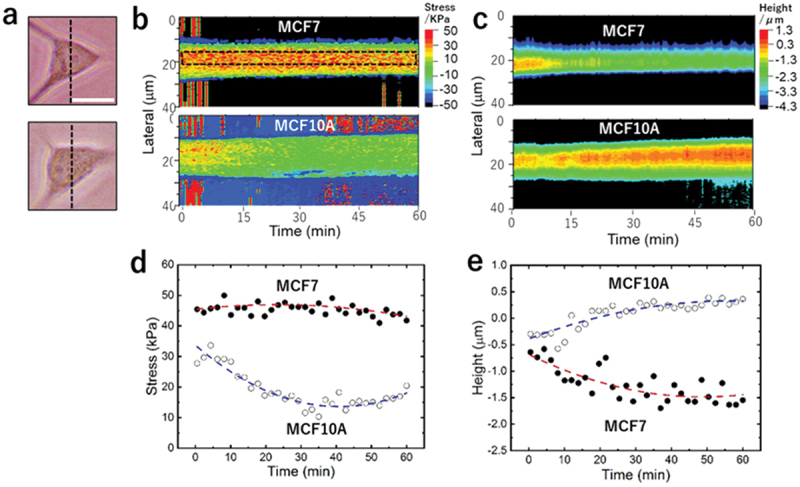
Intracellular prestress response to compressional stimuli for cancerous and normal cells. (a) Phase contrast images of a cancer cell (MCF7, upper) and a normal cell (MCF10A, lower). Scale bar: 20 μm. (b) Time sequences of stacked prestress line profiles collected along dashed lines in (a). (c) Stacked topography profiles collected simultaneously with (b). (d) Time sequence of average prestress values calculated from dashed rectangle region in (b). Height values collected from the same data sequence.

We were motivated to compare force spectrum images of living cells for cancerous and normal types of the same origin. Cell pairs from breast (MCF7 and MCF10A) were both cultured with recommended condition on a plastic dish ready for AFM indentation. After more than 30 attempts, it became clear that while cancer cells could be spectrum-imaged with high success rate (>80%) without cells detaching from substrate, tested normal cells could never remain on substrate during AFM indentation scanning, despite the same AFM operation parameters used. The normal cell detachment, presumably due to cell death, appeared to be caused by mechanical stimulation from the AFM indentation. To better quantify such response differences between cancerous and normal cells, we designed the following experiment. As shown in the optical microscope images of Figure 4(a), a MCF7 cell (upper cell) and a MCF10A cell (lower cell) with similar morphology were selected. In the middle positions of each cell along dashed lines in Figure 4(a), line scans of AFM indentation were continuously repeated for more than 1 hour with repetition time of 30 s. Similar to the mapping (2D scan) situation, there was no noticeable changes on morphology of the MCF7 cell. For each repetition, line profiles of stress and topography could be generated. Such line profiles were stacked according to their capturing time to form a time-sequence of prestress profiles (Figure 4(b)) and a time-sequence of topography profiles (Figure 4c). Figure 4(d) shows the time profiles of the averaged stress within the central 5 μm region of the line scan. While prestress on the MCF7 cancer cell remained practically unchanged throughout the 1-hour measurement period, that on the MCF10A normal cell decayed to a plateau with 50% of initial value in the first 30 minutes. Several MCF10A cells from the same culture dish were tested using this method in turn at times separated by more than a few hours. The very similar behavior of these cells confirmed that the observed cell detachment was due to the inherited response of MCF10A to mechanical stimuli rather than experimental conditions. Figure 4(e) is a plot of height with respect to time. It showed that height values of cancer cell reduced by 85% while height values of normal cell increased by 75%. It might indicate that normal cells got detached from the substrate gradually. The applied indentation force was 3 nN and cell interaction area is ~0.36 μm2, which equates 10 kPa pressure and occupies less than 1% of entire cell surface. We expected that these small mechanical interactions would not cause cell death based on the previous report on which a much larger magnitude of compression (>100 kPa hydrostatic pressure or > 10 μN as total force) is necessary to induce passive necrosis [23]. However, our study revealed a higher sensitivity to mechanically induced cell death for normal cells compared to cancer cells. Therefore, cellular detachment and necrosis process are initiated by compressing an AFM tip on normal cell surface may serve to maintain organ homeostasis [24]. On the other hand, cancer cells are changed not only to survive the compression in tumor microenvironment but also to promote cellular metastasis [25].

### 2.4. Intracellular force distribution is changed through actin relocation during migration initiation

**Figure 5.**
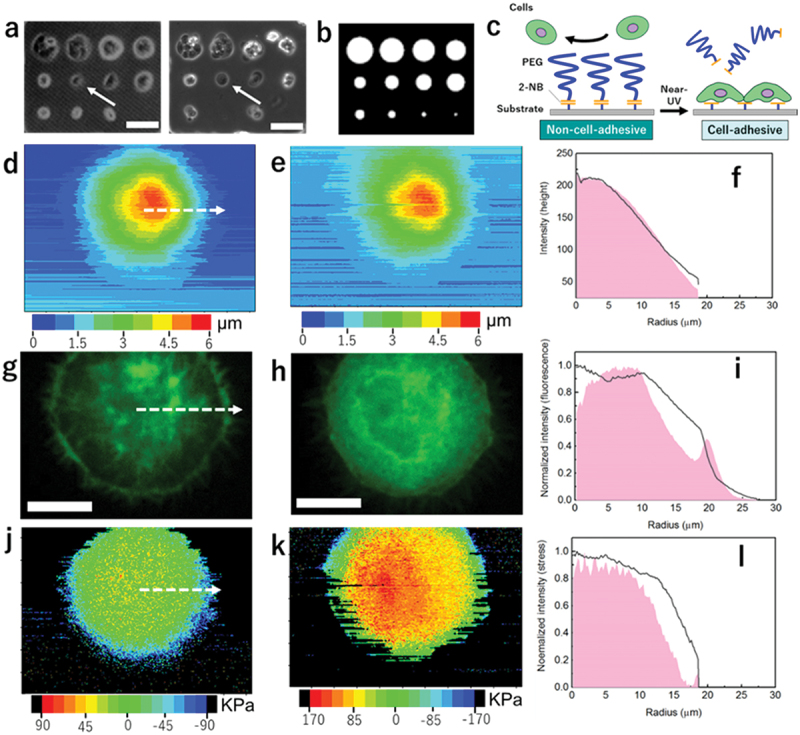
Intracellular prestress distribution upon migration initiation of a stationary cell. (a) Phase contrast images of MDCK cells patterned in given geometrical confinements before (left) and after (right) the confinement release by the secondary irradiation. Scale bar: 50 μm. (b) A pattern of a photomask which black parts were confined. (c) Schematic drawing of the working principle of the photoactivatable surface. (d, e) AFM topography maps collected before and after migration confinement removal. (f) radial distribution profiles measured from (d) (pink) and (e) (black). (g, h) Fluorescence images of actin cytoskeleton in the MDCK cells collected before and after migration confinement removal. Scale bar: 10 μm (i) Radial distribution profiles measured from (g) (pink) and (h) (black). (j, k) Prestress maps collected simultaneously with (d) and (e). (l) Radial distribution profiles measured from (j) (pink) and (k) (black). White dotted arrow in d,g,j indicated the center and radius used for creating such radial profiles.

Finally, we went on to investigate how cells manipulate internal prestress distribution in response to geometrical confinement. For this purpose, we utilized a photoactivatable glass substrate bearing poly (ethylene glycol) (PEG) via photocleavable 2-nitrobenzyl (2-NB) group (Figure 5(a)) [26]. This surface changes from non-cell adhesive to cell adhesive by illumination of light; thereby, we can initially confine the single cells or cell clusters in any geometry (Figure 5(a)), corresponding the pattern of a photomask (Figure 5(b)), and release the confinements by secondary irradiating the remaining regions (Figure 5(c)). AFM spectrum images were taken on a selected cell (white arrow in Figure 5(a)) first in its confined state and 1 hour after it was set in free state by second photo irradiation. The two generated height maps were displayed in Figure 5(d,e). The similarity between these two images suggests that the cell has not begun migration yet, 1 hour after constraint removal. Taking advantage of the circular cell shape, we were able to create height radial distribution for the two images. White arrow in Figure 5(d) indicated the center and radius used for creating such radial profiles. The good match between the two profiles, shown in Figure 5(f), verified that lamellipodia or filopodia has not been formed for the freed cell. The MDCK cell used in this study stably express lifeact-GFP, which allows fluorescence imaging its actin cytoskeleton in a living state. In the confined state, the cell in Figure 5(g) clearly showed a typical actin belt structure that defines boundary for a stationary cell [27]. In the released state as shown in Figure 5(h), on the contrary, cell actin belt disappeared and a dendritic actin network was found in the cytoplasm near the previous cell boundary. Normalized radial distribution profiles made for the two images (Figure 5(i)) showed a peak structure corresponding to actin belt in the constrained state and emergence of new actin network in the freed state. Prestress maps taken on the two cell states were displayed in Figure 5(j,k). Overall tensile stress in the freed cell was found increased. To compare prestress distribution differences, we took normalized radial profiles, shown in Figure 5(l). It is noted that although actin belt shows a strong intensity in the fluorescence image, indicating high actin content, no appreciable tensile stress was generated along the belt. However, significantly increased stress was found on the newly formed actin network region. It was known that cells convert actomyosin bundles from cortical belt into dendritic network to promote motility [28]. The disappearance of actin belt is a hallmark for lamellipodia formation. Our prestress measurement showed evidence that the force-generating capability between the two states of actin bundles are distinctively different. It is speculated that the force generated by the actomyosin network not only help to extrude cytoplasm outward forming lamellipodium, but also help to promote more dendritic actin formation by consuming actin stored previously in the boundary belt [29].

## 3. Materials and methods

### 3.1. Cell cultures

Hela cell line, A549, MDCK and MCF7 cell line were purchased from RIKEN BRC. Cervical primary cell line, lung primary cell line and MCF10A cell line were purchased from American Type Culture Collection (ATCC). Culture media used were: MEM (Thermo Fisher Scientific) with 10% calf serum (CS, Thermo Fisher Scientific) for Hela cell line; DMEM (low glucose, Thermo Fisher Scientific) with 10% fetal bovine serum (FBS, Thermo Fisher Scientific) for A549 cell line; MEM (Thermo Fisher Scientific) with 10% fetal bovine serum (FBS, Thermo Fisher Scientific), 1 mM Sodium Pyruvate (Thermo Fisher Scientific) and 0.1 mM Non-Essential Amino Acids (NEAA, Thermo Fisher Scientific) for MCF7 cell line; cervical epithelial cell basal medium (ATCC PCS-480-032) supplemented with all contents of a cervical epithelial growth kit (ATCC PCS-080-042) for cervical primary cell line; Airway epithelial cell basal media supplemented with bronchial epithelial cell growth kit (ATCC PCS-300-040) components for lung primary cell lines; base medium (Mammary Epithelial Cell Base Medium, MEBM, Lonza) with a growth kit (Mammary Epithelial Cell Growth Medium, MEGM, Lonza) and 100 ng/ml cholera toxin for MCF10A cell line. All cells were plated onto 35 mm petri dish and incubated at 37°C in a 5% CO_2_ atmosphere for 24 hours before taking out for AFM experiments. For photopatterning, MDCK cells stably expressing lifeact-green fluorescence protein (GFP) [30] were grown in MEM (Sigma-Aldrich, St. Louis, MO, USA) supplemented with 10% heat-inactivated fetal bovine serum (FBS, BioWest, Nuaille, EU), 1% MEM non-essential amino acids (MEM-NEAA, Gibco, NY, U.S.A.), 1% sodium pyruvate (Wako, Japan), 1% L-glutamine (Wako, Japan) and 1% penicillin-streptomycin (Wako, Japan) and maintained at 37°Cwith 5% CO_2_.

### 3.2. Photoactivatable surfaces and photo-patterning

The preparation of cell non-adhesive glass surfaces using the photoactivable PEG was performed as previously reported [31] In short, the photocleavable 1-[5-methoxy-2- nitro-4-(trimethoxysilylpropylphenyl)]ethyl N-succinimidyl carbonate is covalently bound to a glass cover (Matsunami φ = 0.17 mm, cleaned with UV-Ozone). Then PEG monoamine (PEG12k-NH) is further coupled to activated glass surfaces. Photopatterning is done by the use of a photomask to allow the attachment of cells in circular patterns with various diameter. Olympus inverted microscope (IF81-PAFM, Olympus, Tokyo, Japan) equipped with an HBO mercury arc lamp (Olympus) and focusing UV light (365 nm) through an 10X objective (UPlanS Apo) was used. Energy of photoactivation is 20 J/cm^2^. After photoactivation, MDCK cells were seeded and then cultured in 37°C for 10 hrs; then AFM measurement was performed. For second irradiation, an opposite photomask was used to allow the irradiation of area around the cells and protect the cell from UV exposure; then AFM measurement was performed.

### 3.3. Immunofluorescence staining

The cells were fixed with 4% paraformaldehyde solution (Sigma-Aldrich Co. LLC) for 15 minutes at room temperature (RT), followed by a permeabilization step using 0.25% Triton X-100 (Sigma-Aldrich Co. LLC) for 10 minutes at RT. After that, the blocking step was carried out by treatment with 5% bovine serum albumin (BSA, Sigma-Aldrich Co. LLC) for 45 minutes at RT. Rat monoclonal α-Tubulin antibody (clone YL1/2, Abcam, Ab6160) diluted 1: 1000 in 1% BSA and incubated with cells for overnight at 4°C. Alexa Fluor Plus 555 Phalloidin (1:50 dilution, Thermo Fisher Scientific), Hoechst 33,342 (1:2000 dilution, Thermo Fisher Scientific), and Alexa Fluor-488 anti-mouse IgG (1:400 dilution, Thermo Fisher Scientific), Alexa Fluor-488 anti-Rat IgG (1:1000 dilution, Life Technologies, Eugene, OR, U.S.A.) were incubated with cells for 30 minutes at RT. A washing step was performed in between all previous steps using phosphate-buffered saline (PBS, Thermo Fisher Scientific) 3 times at RT. Confocal images were obtained using confocal laser microscope (TCS SP5, Leica Microsystems). The observation of the actin-GFP cytoskeleton was performed before and after the photoirradiation using Olympus microscope (IF81-PAFM, Olympus, Tokyo, Japan) equipped with a disk-scan unit (CSU-10, Yokogawa, Tokyo, Japan), and Andro CCD camera (SONA 4BV6U, UK). The actin and MTs were stained, and the Z-stack series of MCF-7 was captured using the same confocal laser scanning microscope. An orthogonal (side) view of MCF-7 cells was obtained using Fiji for cells cultured in a glass-bottom dish for nearly 15 h.

### 3.4. AFM indentation

Cell culture dishes were taken out of incubator and directly characterized by AFM using a liquid probe hand. Gold-coated cantilevers with pyramidal tips (PPP-CONTSCAuD, NANOSENSORS) with spring constant in range of 0.01–1.87 N/m were mounted on the AFM liquid probe hand. Spring constant was calibrated using thermal noise method before every experiment. At each indentation location, tip pressed into the cell until a set force threshold value was reached. Threshold for mapping was set at 3 nN, while that for line profile was 1.5 nN to minimize influence on normal cells.

### 3.5. Calculation of prestress and modulus

We considered real tip shape’s deviation from an ideal hemisphere by treating tip radius *R* as a linear combination of a constant term *r* and specimen dependent terms, which are topographic gradient in *x* direction, *δz/δx*; gradient in y direction, *δz/δy*; and deformation, *d*. The format of *r+aδz/δx+bδz/δy+cd* is adopted. Coefficients *a, b, c, d* were then generated by Markov-chain Monte Carlo method to provide a trial *R* value. Prestress *σ* and modulus *E* were then best fitted using equation (1) at each indentation location during one map scan. Fitting errors at each indentation were also calculated and their variance from the entire map could be obtained. The variance was then used as feedback to the Monte Carlo algorithm for generating the next combination of *a, b, c, d* for 1000 loops till minimization of variance is achieved. The resulting *R* is considered to be the true radius of tip during the map acquisition, because location dependency of fitting error should be minimum. The *σ* and *E* obtained with this *R* value are used to produce the *σ* and *E* maps used for analysis.

## 4. Conclusion

Biochemical exploration of cell biology has reached a level that enables elucidating roles of single molecules functioning in multiple global cascade pathways during cellular response to external stimuli [32]. Though the significance of the involved changes of cellular mechanical property has long been recognized, cellular mechanics characterization has remained coarse grained with low spatial resolution and dependent on over-simplified biophysical models. To fill in this gap, we adopted a machine learning approach to analyze force curves produced by sharp probe non-invasive AFM indention. It enabled lateral prestress existing in living cells to be mapped with nanometric details. We revealed that both cancer cells and normal cells manipulate cellular force distribution in response to stimuli from environment. This maneuver capability could be used by cells toward either a survival by mitigating negative influence from sudden micro-Newton-level external force exertion or cell death after receiving mechanical signals as low as nano-Newton level. It is also found that the active cellular force distribution changes could be implemented either through activating alternative force-generation machinery (nucleoskeleton supplementing impaired cytoskeleton) or converting between different configurations of the same machinery (actomyosin bundles). Following the demonstrated examples and power of this new characterization technique, future development should be further improving force sensitivity toward single molecule force measurement. It would enable direct understanding of mechanical roles for each molecular component in the cell response circuitry, when combining the technique with state-of-the art molecular imaging technologies. In respect of application in the field of tissue engineering and biomaterials, learning about cells’ response to a certain chemical/mechanical cue is of pivotal importance. In principle, any cellular response is first driven by intracellular forces. The described capability to accurately measure such forces in a non-invasive way would speed up evaluation and searching processes toward the most suitable material design [33].

## Supplementary Material

Supplemental Material

## References

[1] Fulda S, Gorman A, Hori O, et al. Cellular stress responses: cell survival and cell death. Int J Cell Biol. 2010;2010:214074. doi: 10.1155/2010/214074 20182529 PMC2825543

[2] Humphery J, Dufresne E, Schwartz M. Mechanotransduction and extracellular matrix homeostasis. Nat Rev Mol Cell Biol. 2014;15:802–11. doi: 10.1038/nrm3896 25355505 PMC4513363

[3] Ingber D, Tensegrity I. Cell structure and hierarchical systems biology. J Cell Sci. 2003;116:1157–1173. doi: 10.1242/jcs.00359 12615960

[4] Guo M, Ehrlicher A, Jensen M, et al. Probing the stochastic, motor-driven properties of the cytoplasm using force spectrum microscopy. Cell. 2014;158(4):822–832. doi: 10.1016/j.cell.2014.06.051 25126787 PMC4183065

[5] Meng F, Sachs F. Visualizing dynamic cytoplasmic forces with a compliance-matched FRET sensor. J Cell Sci. 2010;124:261–269. doi: 10.1242/jcs.071928 21172803 PMC3010192

[6] Gonzále M, Latorre E, Arroyo M, et al. Measuring mechanical stress in living tissues. Tech Rev. 2020;2:301–317. doi: 10.1038/s42254-020-0184-6 PMC7617344 39867749

[7] Garcia R. Nanomechanical mapping of soft materials with the atomic force microscope: methods, theory and applications. Chem Soc Rev. 2020;49:5850–5884. doi: 10.1039/D0CS00318B 32662499

[8] Mandriota N, Friedsam C, Molina J, et al. Cellular nanoscale stiffness patterns governed by intracellular forces. Nat Mater. 2019;18(10):1071–1077. doi: 10.1038/s41563-019-0391-7 31209386 PMC6754298

[9] Zemła J, Bobrowska J, Kubiak A, et al. Indenting soft samples (hydrogels and cells) with cantilevers possessing various shapes of probing tip. Eur Biophys J. 2020;49(6):485–495. doi: 10.1007/s00249-020-01456-7 32803311 PMC7456413

[10] Reischl B, Rohl A, Kuronen A, et al. Atomistic simulation of the measurement of mechanical properties of gold nanorods by AFM. Sci Rep. 2017;7(1):16257. doi: 10.1038/s41598-017-16460-9 29176635 PMC5701227

[11] Muller M, Geiss R, Hurley D. Contact mechanics and tip shape in AFM-based nanomechanical measurements. Ultramicroscopy. 2006;106(6):466–474. doi: 10.1016/j.ultramic.2005.12.006 16448755

[12] Mackey D, Hogg D, Lang D, et al. emcee: the MCMC hammer, Publ. Astron Soc Pac. 2013;125:306–312. doi: 10.1086/670067

[13] Hertz H. Über die Berührung fester elastischer Körper. J Fur Reine Angew Math. 1881;92:156–171.

[14] Sneddon L. The relation between load and penetration in the axisymmetric boussinesq problem for a punch of arbitrary profile. Int J Eng Sci. 1965;3:47–57. doi: 10.1016/0020-7225(65)90019-4

[15] Polop C, Vasco E, Perrino A, et al. Mapping stress in polycrystals with sub-10nm spatial resolution. Nanoscale. 2019;9:13938–13946. doi: 10.1039/C7NR00800G 28686260

[16] Rosenbluth M, Lam W, Fletcher D. Force microscopy of nonadherent cells: a comparison of leukemia cell deformability. Biophys J. 2006;90(8):2994–3003. doi: 10.1529/biophysj.105.067496 16443660 PMC1414579

[17] Garcia P, Garcia R. Determination of the elastic moduli of a single cell cultured on a rigid support by force microscopy. Biophys J. 2018;114(12):2923–2932. doi: 10.1016/j.bpj.2018.05.012 29925028 PMC6026379

[18] Wang H, Zhang H, Da B, et al. Mechanomics biomarker for cancer cells unidentifiable through morphology and elastic modulus. Nano Lett. 2021;21(3):1538–1545. doi: 10.1021/acs.nanolett.1c00003 33476166

[19] Santos A, Cook A, Gough R, et al. DNA damage alters nuclear mechanics through chromatin reorganization. Nucleic Acids Res. 2021;49(1):340–353. doi: 10.1093/nar/gkaa1202 33330932 PMC7797048

[20] Guilluy C, Osborne L, Landeghem L, et al. Isolated nuclei adapt to force and reveal a mechanotransduction pathway within the nucleus. Nat Cell Biol. 2014;16(4):376–381. doi: 10.1038/ncb2927 24609268 PMC4085695

[21] Shivashankar G. Mechanosignaling to the cell nucleus and gene regulation, Annu. Rev Biophys. 2011;40:361–378. doi: 10.1146/annurev-biophys-042910-155319 21391812

[22] Amar K, Wei F, Chen J, et al. Effects of forces on chromatin. APL Bioeng. 2021;5(4):041503. doi: 10.1063/5.0065302 34661040 PMC8516479

[23] Valon L, Levayer R. Dying under pressure: cellular characterisation and in vivo functions of cell death induced by compaction. Biol Cell. 2019;111(3):51–66. doi: 10.1111/boc.201800075 30609052

[24] Proskuryakov S, Gabai V, Konoplyannikov A. Necrosis is an active and controlled form of programmed cell death. Biochemistry (Moscow). 2002;67(4):387–408. doi: 10.1023/A:1015289521275 11996653

[25] Tse J, Cheng G, Tyrrell J, et al. Mechanical compression drives cancer cells toward invasive phenotype. PNAS. 2019;109(3):911–916. doi: 10.1073/pnas.1118910109 PMC3271885 22203958

[26] Nakanishi J, Kikuchi Y, Inoue S, et al. Spatiotemporal control of migration of single cells on a photoactivatable cell microarray. J Am Chem Soc. 2007;129(21):6694–6695. doi: 10.1021/ja070294p 17488076

[27] Abdellatef S, Nakanishi J. Photoactivatable substrates for systematic study of the impact of an extracellular matrix ligand on appearance of leader cells in collective cell migration. Biomaterials. 2018;169:72–84. doi: 10.1016/j.biomaterials.2018.03.045 29655082

[28] Lomakin A, Lee K, Han S, et al. Competition for actin between two distinct F-actin networks defines a bistable switch for cell polarization. Nat Cell Biol. 2015;17(11):1435–1445. doi: 10.1038/ncb3246 26414403 PMC4628555

[29] Rausch S, Das T, Soiné J, et al. Polarizing cytoskeletal tension to induce leader cell formation during collective cell migration. Biointerphases. 2013;8(1):32. doi: 10.1186/1559-4106-8-32 24706149

[30] Chang A, Uto K, Homma K, et al. Viscoelastically tunable substrates elucidate the interface-relaxation-dependent adhesion and assembly behaviors of epithelial cells. Biomaterials. 2021;274:120861. doi: 10.1016/j.biomaterials.2021.120861 33991949

[31] Rolli C, Nakayama H, Yamaguchi K, et al. Switchable adhesive substrates: revealing geometry dependence in collective cell behavior. Biomaterials. 2012;33(8):2409–2418. doi: 10.1016/j.biomaterials.2011.12.012 22197568

[32] Chandarlapaty S. Negative feedback and adaptive resistance to the targeted therapy of cancer. Cancer Discov. 2012;2(4):311–319. doi: 10.1158/2159-8290.CD-12-0018 22576208 PMC3351275

[33] Koons G, Diba M, Mikos A. Materials design for bone tissue engineering. Nat Rev Mater. 2020;5:584–603. doi: 10.1038/s41578-020-0204-2

