# Supplementary material for: Mapping stress inside living cells by atomic force microscopy in response to environmental stimuli
Source: Sci Technol Adv Mater. 2023 Oct 18;24(1):2265434. doi: 10.1080/14686996.2023.2265434 (PMC10586080; doi:10.1080/14686996.2023.2265434)
Supplement: Supplemental Material [file TSTA_A_2265434_SM0316.docx]

Supplementary Information

Mapping stress inside living cells by atomic force microscopy in response to environmental stimuli

Hongxin Wang^a^, Han Zhang^a^*, Ryo Tamura^a^, Bo Da^a^, Shimaa A. Abdellatef^a^, Ikumu Watanabe^a^, Nobuyuki Ishida^a^, Daisuke Fujita^a^, Nobutaka Hanagata^a^, Tomoki Nakagawa^b^, Jun Nakanishi^a^*

^a^National Institute for Materials Science, Sengen 1-2-1, Tsukuba, Ibaraki, Japan 305-0047; ^b^University of Tsukuba Hospital, Amakubo 2-1-1, Tsukuba, Ibaraki, Japan 305-8576

* To whom correspondence should be addressed: ZHANG.Han@nims.go.jp, NAKANISHI.Jun@nims.go.jp


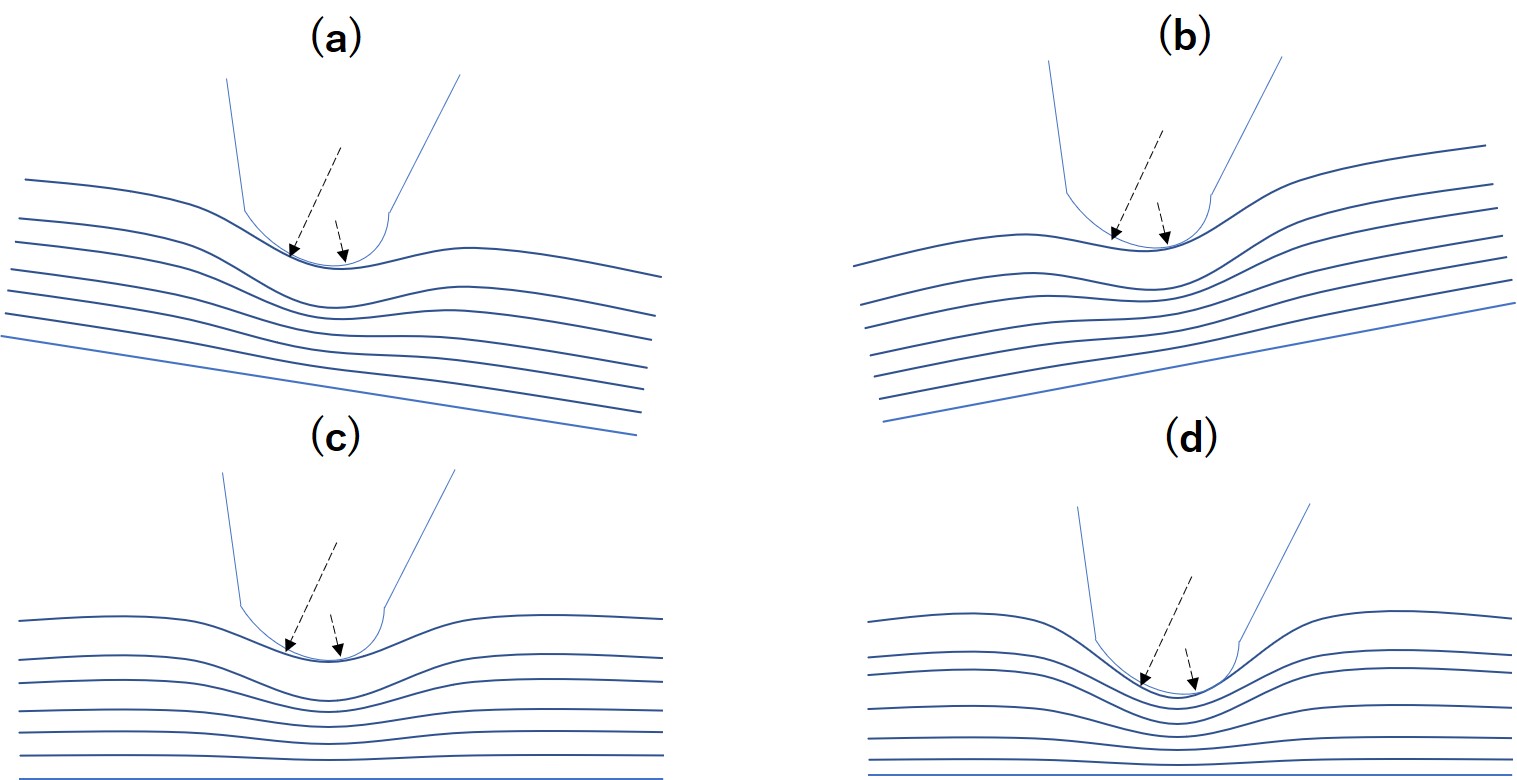


Figure S1: Accurate determination of AFM tip geometry. (a) and (b) representing tip radius dependence on surface topography. (c) and (d) representing tip radius dependence on depth; AFM tip radius R is defined as a function of local gradient and indentation depth: $R=r+a\cdot\delta z/\delta x+b\cdot\delta z/\delta y+c\cdot d$


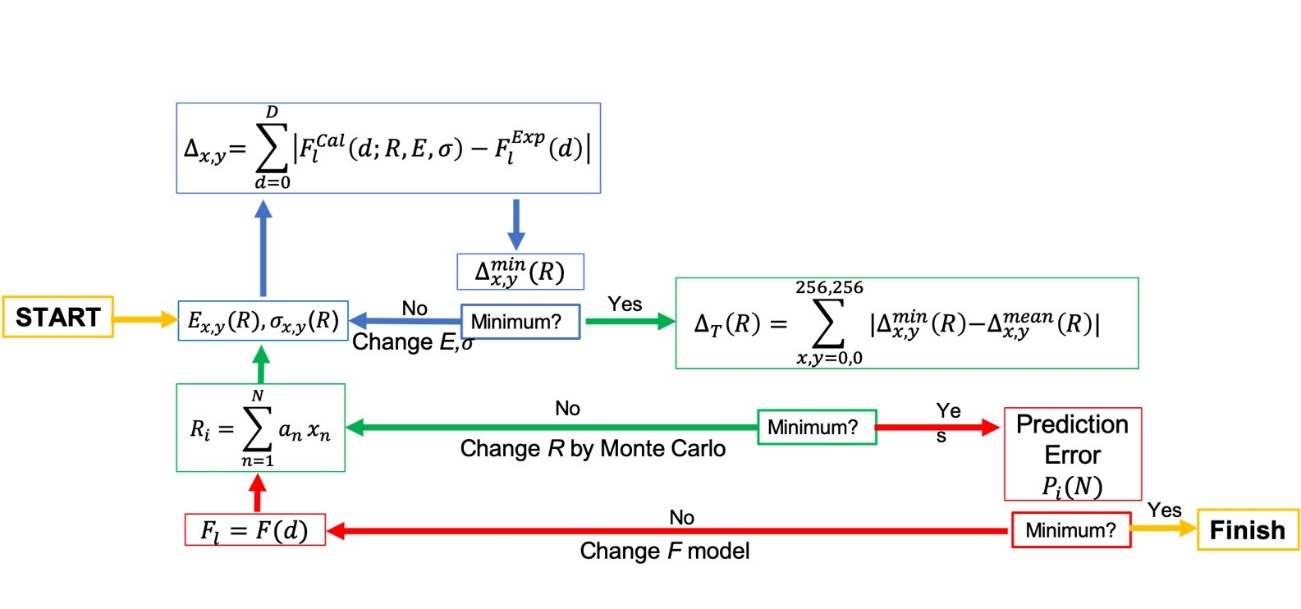


Figure S2. Optimization scheme with 3 layers of iteration to find the most suitable AFM model, tip radius, elastic modulus and stress.


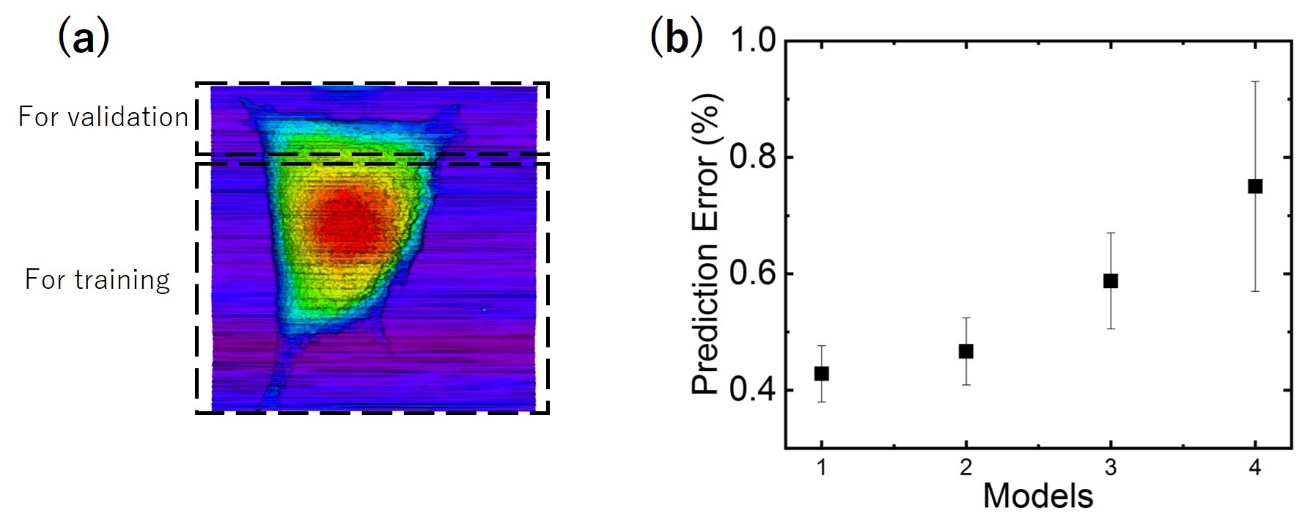


Figure S3: (a) Strategy of calculating prediction errors for each AFM model: 1/8 of data is used for validation while the remaining 7/8 for training. (b) Prediction error comparison for 4 AFM models: 1-4 as in figure 1a.

**
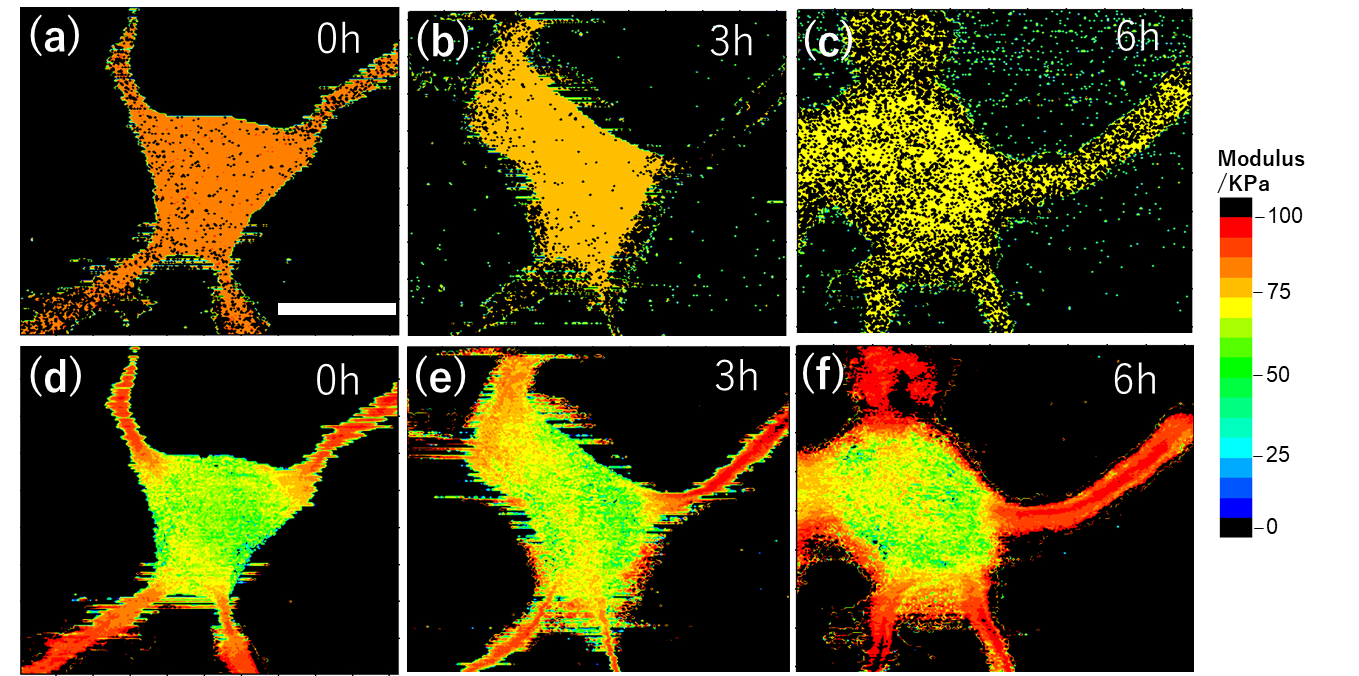
**

Figure S4: (a)-(c) A series of modulus maps of the MCF7 cell produced using indentation curves up to a depth of 100 nm, following a time sequence after Myosin II inhibition. Modulus data collected on cell cortex. Scale bar: 20 μm. (d)-(f) Same type of modulus maps using full depth of 800 nm. Modulus data collected on cell nucleoskeleton.


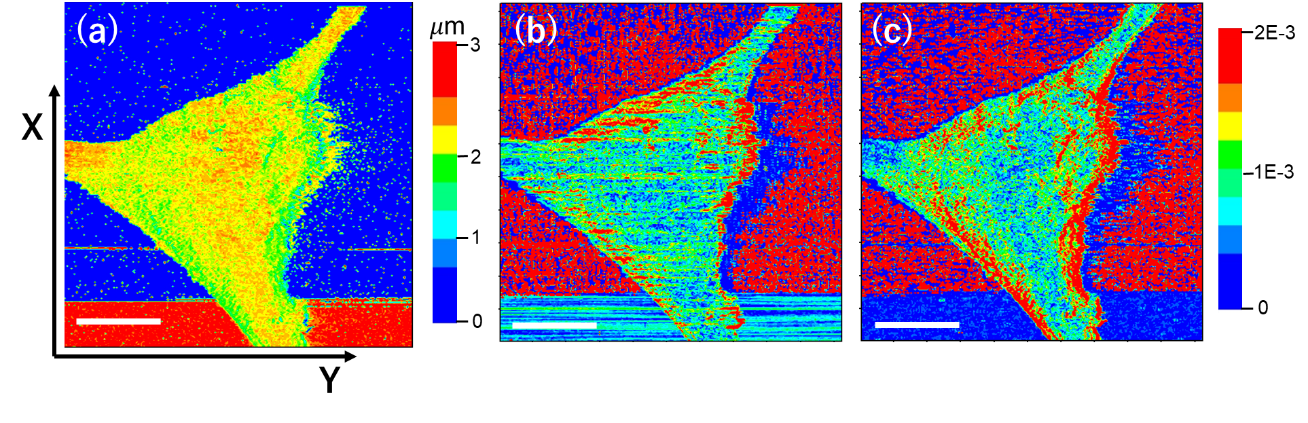


Figure S5: (a) Heat map for *R* value with full indentation depth after MCMC optimization; (b) and (c) Topography gradient maps in the x direction and y direction. Scale bar: 10um.


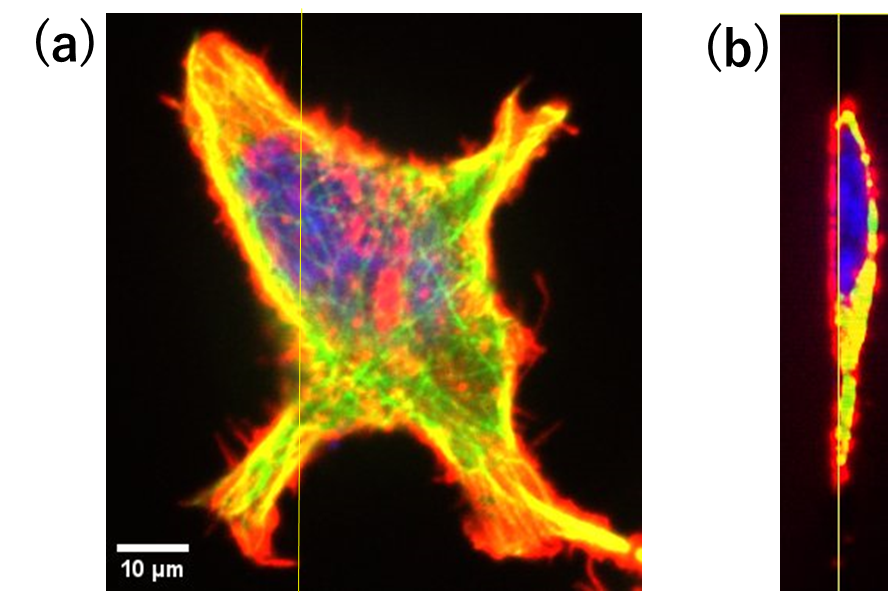


Figure S6: (a) Top view of a typical confocal fluorescence image of a MCF7 cell, with colors of blue, green and red representing nucleus, microtubule and actin fiber; (b) Cross-section view at a position marked by a vertical line in (a).


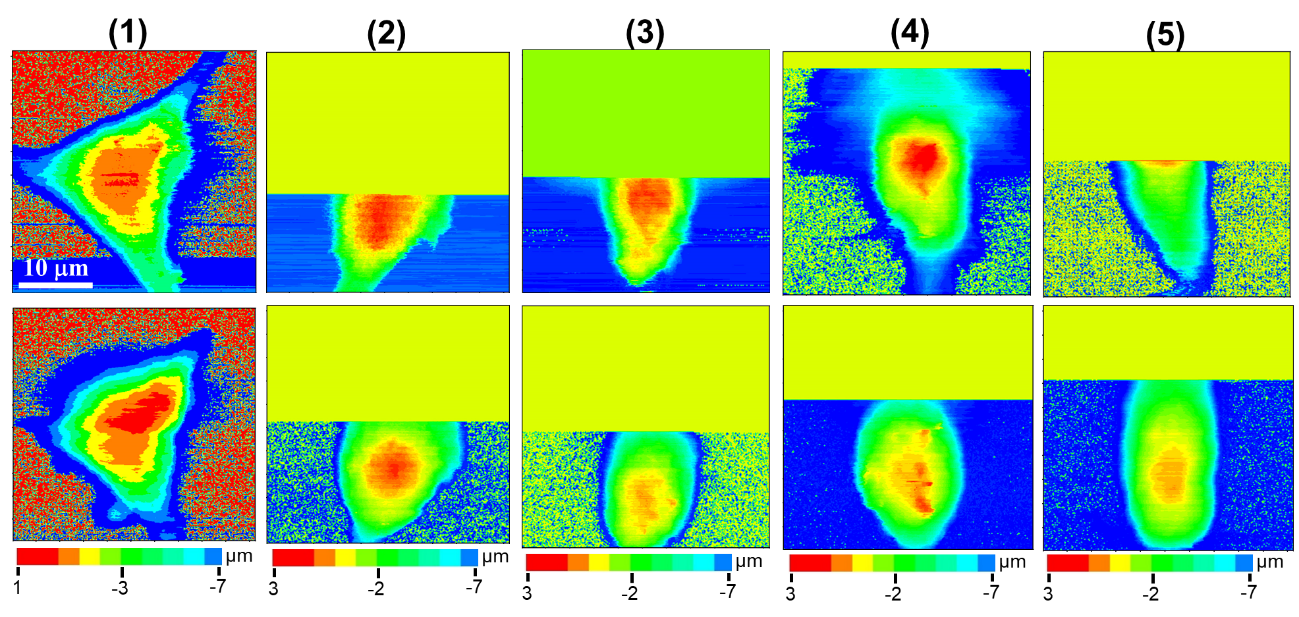


Figure S7: Topography maps of 5 cells before (upper row) and after (lower row) PFA fixation.


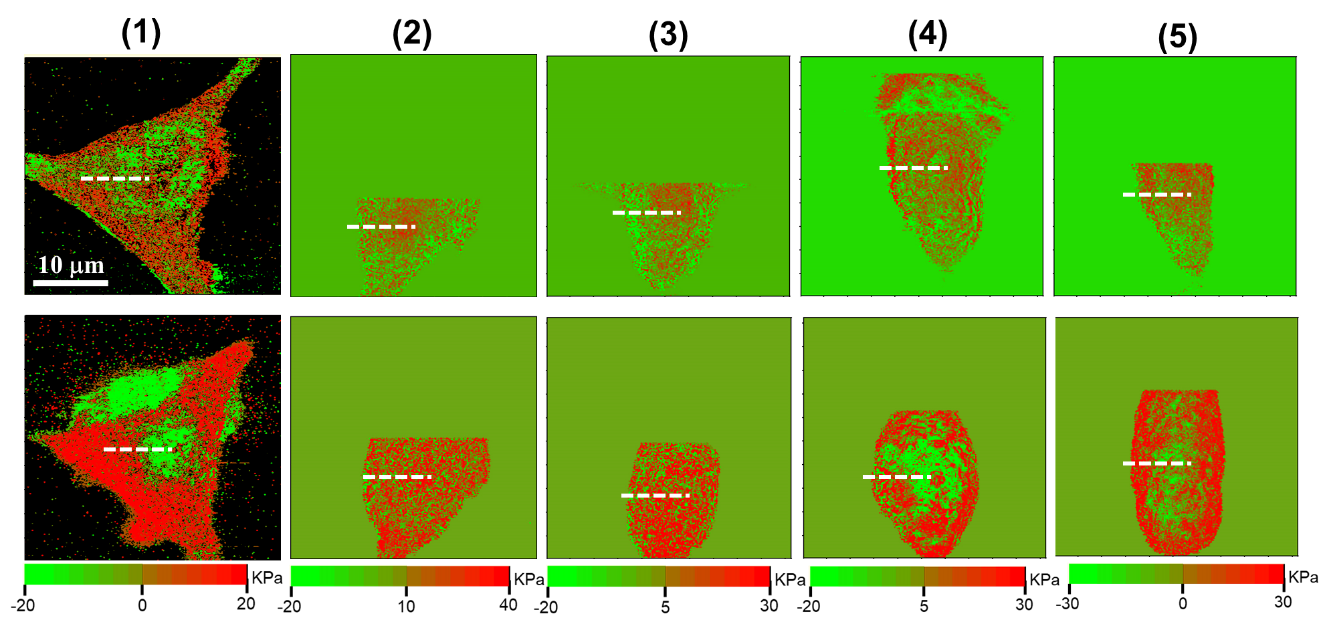


Figure S8: Stress maps of 5 cells before (upper row) and after (lower row) PFA fixation.


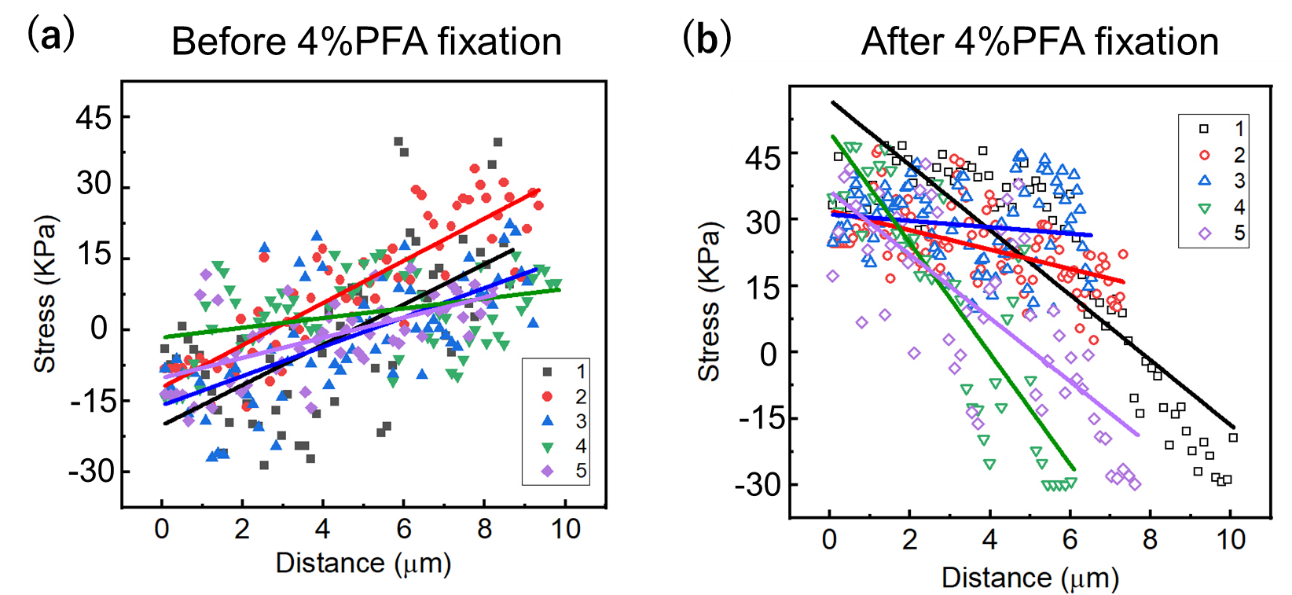


Figure S9: Stress line profiles acquired along dashed lines in figure S8 for living cells (a) and their fixed cell counterparts (b).

we performed extra 4 sets of experiments to compare stress images of a living cell and a fixed cell. (a same cell before and after PFA fixation) Except for the first fully-scanned cell, we scanned a bit over half cell area to ensure enough data for analysis and avoid possible cell detachment during scanning. Their topography maps are displayed with upper row as living cells and lower row as fixed cells, in figure S7 of supplementary information. The corresponding stress maps are displayed in figure S8. Both living cells and fixed cells presented similar topography features with highest point being nucleus region. This similarity ensured that topography influence on AFM data acquisition is equal for both cell types. However, stress maps showed opposite distribution: living cells are with tensional stress in the nucleus region and it transits into compressional stress towards cell edge; while compressional stress are often found on nucleus in their fixed counterparts with transition into tensional type near cell edge. A line profile (white lines in images) from cell edge to nucleus was plotted for each cell to better quantify this trend. (figure S9) Trend lines showed positive slopes for all living cells and negative slopes for all their fixed cell counterparts. In conclusion, tensional stress distribution in living cell nucleus region and their changes towards compressional stress after chemical fixation have been confirmed.


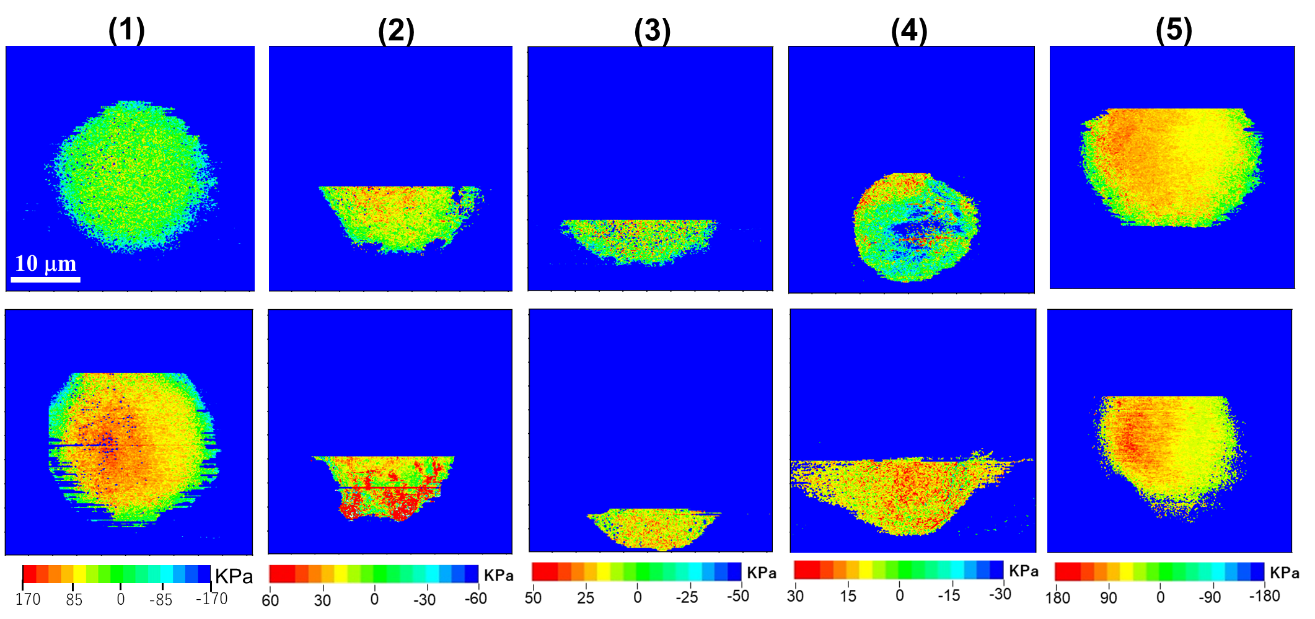


Figure S10: Stress maps of 5 cells before (upper row) and after (lower row) migration confinement removal through UV irradiation.


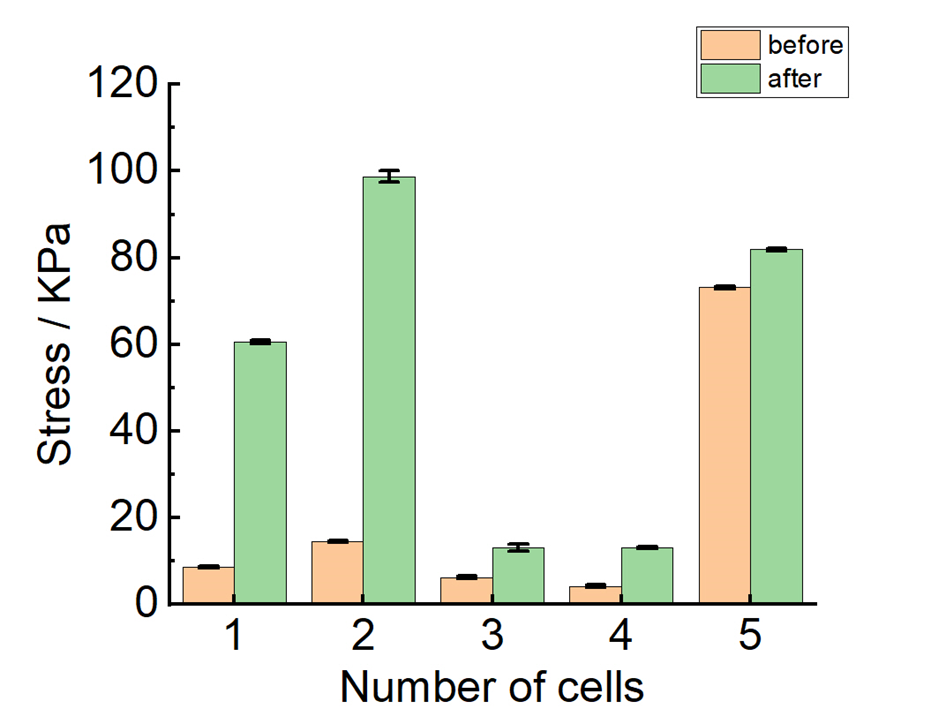


Figure S11: Bar chart showing stress values averaged over scanned cell surface for the 5 cells in figure S10.

5 living cells on a pattern-confined substrate were scanned for stress maps before and after their confinement removal through UV irradiation. In this set of experiment, though most cells were scanned only partially, we intended to compare stress values averaged over scanned cell surface area. (See figure S10) Statistical result is presented in a bar chart of figure S.11 (supplementary information). All cells exhibited an enhanced tensional stress level after their migration confinement was removed by UV radiation. The repeatability of the observation was thus confirmed.
